# Balancing the risks and benefits of antibiotic use in a globalized world: the ethics of antimicrobial resistance

**DOI:** 10.1186/s12992-023-00930-z

**Published:** 2023-04-20

**Authors:** Yusuff Adebayo Adebisi

**Affiliations:** 1 Nuffield Department of Population Health, University of Oxford, Oxford, UK; 2 Global Health Focus, Kigali, Rwanda

**Keywords:** Antimicrobial resistance, Antibiotics, Ethics, Equity, Antimicrobial stewardship

## Abstract

Antimicrobial resistance (AMR) is a “silent pandemic” that threatens the efficacy of antibiotics and other antimicrobials. It is imperative to take into account the ethical implications of how these resources are used and distributed as the world deals with this silent pandemic. This commentary discusses the ethical considerations surrounding the use and distribution of antibiotics in the age of resistance, including issues of equity and access, responsibility for antimicrobial stewardship, the environmental impact of antibiotic use, and the development and promotion of these drugs. The ethical implications of unequal access to antibiotics and the role of social determinants of health in shaping this access are considered, as well as the globalization of AMR and the need for multiple stakeholders to be involved in addressing this issue. The opportunities of antimicrobial stewardship programmes for optimising antibiotic use and reducing the emergence and spread of resistant bacteria, as well as the ethical implications of implementing such programmes, are examined. The potential environmental outcomes of antibiotic use and the ethical implications of these impacts are also discussed, as well as the role of the pharmaceutical industry in the development and promotion of these drugs, the potential conflicts of interest that may arise and the ethical dimension of resource transfer from Global North to Global South. This paper emphasises the significance of a holistic strategy to AMR that considers these ethical components, as well as the importance of preserving antibiotic efficacy for future generations.

## Introduction

Antimicrobial resistance (AMR) is a major global public health concern that has the potential to undermine the clinical usefulness of antibiotics and other antimicrobial drugs [1–3]. Antimicrobials are used to treat a number of infections caused by bacteria, viruses, fungi, and other microorganisms, and their effectiveness is essential for maintaining public health and well-being [2]. However, when pathogens evolve to survive exposure to antimicrobials, it can lead to the spread of drug-resistant infections [1, 2]. AMR is estimated to cause at least 700,000 deaths annually [4]. This number is expected to increase unless action is taken to address the problem. AMR is driven by a variety of factors, including but not limited to the overuse and misuse of antibiotics, poor infection control practices, and inadequate investment in research and development [2, 5]. It is a complex issue that requires a multifaceted approach to address, including efforts to optimize the use of antibiotics, improve infection control practices, and invest in research and development. A collaborative effort and a comprehensive approach are essential in preserving the effectiveness of antibiotics and safeguarding public health for future generations.

The ethical aspects of antibiotic use and distribution are crucial to address when confronting the global challenge of AMR. Antibiotic ethics encompass the ethical considerations related to the use and distribution of these medications, touching on matters of equity, access, responsibility, and environmental impact. In the era of resistance, these concerns gain particular significance, as the improper and excessive use of antibiotics can lead to the development and dissemination of resistant bacteria. This commentary delves deeper into the ethical considerations associated with antibiotic use and distribution in the age of resistance. I underscore the importance of adopting a comprehensive approach to tackle AMR, one that considers these ethical dimensions and stresses the need to maintain the effectiveness of antibiotics for future generations. Additionally, I will explore the ethical ramifications of unequal access to these medications, the responsibility involved in fostering antimicrobial stewardship, the environmental consequences of antibiotic consumption, and the development and marketing of these drugs as well as ethical dimension of resource transfer.

## Antimicrobial resistance and the ethics of antibiotic use

The ethical principle of nonmaleficence, which mandates that harm should not be caused, is a foundational concept in medicine. Antibiotic use can lead to unintended consequences, such as the emergence of resistance, necessitating a careful evaluation of the potential advantages and drawbacks of using these medications [6]. This involves thoroughly assessing the risks and benefits of antibiotic use in various situations and implementing risk-reduction strategies, including programs dedicated to responsible antimicrobial stewardship. Another integral ethical aspect is the principle of beneficence, which obliges us to act in ways that promote good outcomes. Antibiotics serve as a crucial resource for preserving lives and enhancing health, making it imperative to utilize them judiciously to ensure their continued effectiveness [2]. This entails a thoughtful examination of the potential advantages of antibiotic use and the adoption of strategies aimed at optimizing these benefits, such as initiatives that foster responsible antimicrobial stewardship. By upholding these ethical principles and implementing stewardship programs, we can better navigate the complexities of antibiotic use in a world grappling with AMR.

In accordance with the principle of autonomy, individuals are entitled to make informed decisions about their health, including accessing antibiotics when genuinely necessary. To uphold this principle, it is pertinent to ensure that people can obtain antibiotics when required, as well as respect their right to make independent health choices. Educating the public on the appropriate use of antibiotics and the potential consequence of misuse is crucial for informed decision-making. However, striking a balance between respecting autonomy and recognizing the importance of antimicrobial stewardship [7] is essential to prevent antibiotic misuse, overuse, and the subsequent rise in AMR. Antimicrobial stewardship programs, which promote the appropriate use of antibiotics, can help maintain this balance. By implementing such programs, healthcare providers can encourage responsible antibiotic use without infringing on individuals’ autonomy. These initiatives include shared decision-making between patients and healthcare providers, ensuring that antibiotics are only prescribed when necessary, and monitoring antibiotic use to identify and address patterns of misuse.

In line with the principle of justice, the benefits and burdens of antibiotic use should be fairly distributed among individuals and communities. This principle involves addressing access and affordability concerns, such as implementing policies to provide essential antibiotics at low or no cost to those in need and allocating resources for the research and development of new antibiotics [7]. Additionally, it necessitates tackling issues of equity and social determinants of health that may impact access to these medications, such as investing in healthcare infrastructure in underserved areas and reducing disparities in healthcare access and quality. Working to address these concerns is essential in creating a healthcare landscape that promotes justice and equity, ensuring that antibiotics are accessible to those who need them while also being used responsibly.

## Equity and access to antibiotics

Equity and accessibility are one of the most important ethical considerations surrounding the use and distribution of antibiotics. These drugs are a scarce resource, so it is essential that they are used fairly [8]. This may involve ensuring that vulnerable populations and communities have access to antibiotics, regardless of their ability to pay or location. People in low-income countries frequently have limited access to antibiotics due to economic barriers, and people in rural areas may have limited access due to a lack of infrastructure and health care facilities. In addition, certain vulnerable populations, such as refugees and migrants, may encounter access barriers to health care and antibiotics due to socioeconomic factors. To ensure equity and accessibility in the use and distribution of antibiotics, practical solutions include implementing policies that remove economic and geographic barriers to access, increasing healthcare infrastructure in rural areas, and developing targeted programs that specifically address the needs of vulnerable populations, such as refugees and migrants. Additionally, education and awareness-raising campaigns can promote responsible use of antibiotics and reduce unnecessary demand, helping to conserve this limited resource for those who need it most.

Significant ethical implications result from unequal access to antibiotics. Inadequate access to these medications can result in the spread of resistant infections, which can eventually compromise the efficacy of antibiotics for everyone. Antibiotic accessibility is not only a moral necessity, but also a practical necessity. In addition to the human suffering caused by insufficient access to these medications, there are also economic repercussions. The World Health Organization estimates that AMR will cost the global economy $100 trillion by 2050 [9]. The influence of social determinants of health on antibiotic access [10] is also an important ethical factor to consider. Social determinants of health are the factors such as income, education, and living conditions that influence the health of individuals and populations. These factors can significantly influence access to health care and the efficacy of interventions, including antibiotic accessibility. Addressing the social determinants of health is essential to ensuring antibiotic access and equity. This may involve improving income, education, and living conditions, as well as increasing access to health care and antibiotics in underserved areas, implying a multifaceted approach that involves policy interventions, community engagement, and healthcare system improvements is much-needed.

The issue of rationing or prioritising access to these drugs is an additional ethical consideration related to equity and antibiotic access. In situations where there is limited access to antibiotics, difficult decisions may need to be made regarding who receives these medications and who does not. This can raise ethical questions regarding fairness, justice, and resource allocation. Access to antibiotics may need to be prioritised based on the severity of illness, the likelihood of success, and the potential benefits and harms to the individual and society [11]. Instances of rationing or prioritising antibiotic access include situations in which a particular drug is in short supply or the demand for antibiotics exceeds the supply. In these circumstances, it may be necessary to determine who has access to the limited antibiotic supply. It may be necessary, for instance, to prioritise access for patients with the most severe infections or those who are most likely to benefit from treatment.

The ethical implications of rationing or prioritizing access to antibiotics must be carefully considered, particularly during a pandemic when healthcare systems are stretched, and pressure-filled decisions must be made. In such situations, healthcare professionals must strike a much-needed balance between rapidly treating patients and avoiding the potential consequences of overprescribing antibiotics. Adherence to clinical guidelines, appropriate use of diagnostic tests, and thorough assessment of risks and benefits in each case are essential components of responsible antibiotic use. The COVID-19 pandemic has laid bare the widespread misuse of antibiotics, emphasizing the urgency of addressing this issue, both now and in preparation for future pandemics [12]. A collaborative approach that involves multiple stakeholders, such as healthcare providers, policymakers, and the public, as well as grounded in science can help ensure more informed and transparent decision-making. By fostering open communication and sharing the criteria used to make these decisions, a more ethically sound and effective approach to antibiotic use during pandemics can be achieved.

## Stakeholders in antimicrobial stewardship

AMR is a global issue that necessitates the collective effort of governments, the pharmaceutical industry, healthcare professionals, and the general public to combat [13]. One Health is a concept that recognizes the interconnection between human, animal, and environmental health. It has become increasingly important in the fight against AMR as the overuse and misuse of antibiotics in both humans and animals can contribute to the development of AMR. One Health approaches can promote collaboration between healthcare providers, veterinarians, and environmental experts to develop effective interventions that promote responsible use of antibiotics and reduce the risk of AMR. Therefore, promoting One Health approaches is essential to promoting a holistic and effective approach to addressing the global threat of AMR.

Each stakeholder has a role to play in promoting antibiotic stewardship and reducing the emergence and spread of resistant bacteria while upholding ethical practices. Governments, in particular, have a critical responsibility in implementing stewardship programs, prescribing guidelines, regulating antibiotic use in agriculture and other sectors, and funding research and development of new treatments [13]. Despite the recognition of the benefits of antimicrobial stewardship programs, more support is needed to effectively implement these programs in countries that have not yet adopted them. Furthermore, the pharmaceutical industry can contribute by investing in research and development to identify new antibiotics and therapies, while also ensuring the accessibility of antibiotics in low-income nations [14]. The general public also has a role in promoting proper hygiene and seeking medical care when necessary to limit the spread of antibiotic-resistant bacteria. It is important to evaluate the ethical implications of different approaches to antimicrobial stewardship, considering their effects on the availability and efficacy of antibiotics and the well-being of individuals and populations.

Effective antimicrobial stewardship necessitates collaboration among various stakeholders, such as healthcare providers, patients, and regulatory bodies. International cooperation and data sharing are also crucial in combating AMR, which demands a coordinated effort from all involved parties. To promote responsible antibiotic use and develop new treatments, stakeholders should collaborate on strategies and share data and resources. Addressing ethical challenges in collaboration, stakeholders must establish clear guidelines for data sharing, protect intellectual property rights, and foster trust.

## Environmental impact of antibiotic use

The improper disposal and use of antibiotics can have a significant impact on ecosystems, particularly on microbial communities and their function [15, 16]. For example, antibiotics can disrupt the natural balance of microbial populations, leading to an increase in harmful bacteria and a decrease in beneficial microbes. The overuse and misuse of antibiotics can also contribute to the emergence and spread of antibiotic-resistant bacteria in the environment, which can pose a significant threat to human health [1, 4, 5, 17]. To mitigate these negative effects, it is important to consider the environmental impact of antibiotic use and promote responsible antibiotic use practices that prioritize the protection of both human and environmental health.

To ensure responsible use of antibiotics, it is important to educate both healthcare providers and the general public about the potential environmental impacts of antibiotic use and the importance of proper disposal. This education should also emphasize the role of individual responsibility in minimizing the environmental impact of antibiotic use, such as reducing unnecessary antibiotic prescriptions, not flushing antibiotics down the toilet, and returning unused antibiotics to pharmacies for proper disposal. Additionally, governments and regulatory agencies can encourage the development and implementation of sustainable practices for antibiotic manufacturing and use in agriculture and other sectors. The ethical use of antibiotics in animal agriculture raises concerns about the environmental impact and the spread of AMR. Overusing antibiotics for growth promotion and disease prevention in livestock can contribute to the development of drug-resistant bacteria, which may ultimately affect human health. As such, it is crucial to balance the demands of the agricultural industry with the need to preserve the efficacy of antibiotics and protect public health. This may include promoting the use of alternatives to antibiotics in agriculture, such as probiotics and prebiotics, as well as implementing regulations on the discharge of untreated wastewater from pharmaceutical manufacturing facilities and hospitals [16]. By taking these steps, we can help preserve the efficacy of antibiotics for future generations while also protecting human and environmental health.

Responsible antibiotic use practices that prioritize the protection of both human and environmental health are essential to mitigate the negative impacts of antibiotic use on ecosystems and public health. This can be achieved through education and awareness campaigns that emphasize individual responsibility, the promotion of sustainable practices for antibiotic manufacturing and use in agriculture and other sectors, and the development of alternative treatments that have fewer environmental impacts and are less likely to contribute to antibiotic resistance.

## Conflict of interest in the development and promotion of antibiotics by the pharmaceutical industry

The development and promotion of antibiotics by the pharmaceutical industry is an important ethical consideration [5, 18, 19], as their role is crucial in ensuring the availability and accessibility of these lifesaving drugs. However, the financial incentives and potential conflicts of interest that may occur in the development and promotion of antibiotics raise ethical concerns regarding the responsibility of these companies to put the public interest ahead of their own profit [20]. A potential source of competing interests in the development and promotion of antibiotics is the profit motive of pharmaceutical industries. The high costs of research and development (R&D) and the financial risks associated with the development of new drugs can hinder their production, especially for smaller or less profitable markets. Instead of focusing on the needs of patients or the public health consequences of antibiotic resistance, pharmaceutical companies may prioritise the development and marketing of more likely to be profitable drugs. This can lead to a lack of investment in the development of new antibiotics or a focus on developing drugs that are not truly necessary, such as those that differ only marginally from existing antibiotics.

This conflict of interest is exemplified by the overproduction of antibiotics with a narrow spectrum, which are designed to target specific types of bacteria. Although narrow-spectrum antibiotics may be effective in certain circumstances, they can also contribute to the development of antibiotic resistance because they are only effective against a subset of bacteria as opposed to a broad spectrum of pathogens. Due to their targeted nature and potential for multiple patent protections, narrow-spectrum antibiotics may be more lucrative for pharmaceutical companies to develop and market [21]. However, the overuse of antibiotics with a narrow spectrum can contribute to the emergence of resistant bacteria and may not meet the needs of patients in the context of broader public health [22]. One possible approach is to incentivize the development of antibiotics that address unmet medical needs, rather than simply focusing on the profitability of narrow-spectrum antibiotics or drugs that offer marginal improvements over existing treatments. Governments could provide financial incentives, such as grants or tax credits, to encourage the development of antibiotics that address critical public health needs.

Another potential conflict of interest in the development and promotion of antibiotics is the pharmaceutical industry’s influence on healthcare providers’ prescribing practices [23]. Marketing and promotional campaigns may be used by pharmaceutical companies to influence the prescribing habits of healthcare providers and increase product sales. Pharmaceutical companies may, for instance, sponsor continuing education programmes or events or provide funding for research studies that may be biased in favour of their products. These practices can influence the decisions of healthcare professionals and lead to the inappropriate or excessive use of antibiotics. One practical solution is to increase transparency and accountability in the relationship between pharmaceutical companies and healthcare providers. This could involve requiring companies to disclose all payments made to healthcare providers for marketing and promotional activities or prohibiting certain forms of marketing that could lead to the inappropriate use of antibiotics.

Another example of this conflict of interest is the promotion of antibiotics for conditions that are not caused by bacterial infections, such as viral infections or allergies. Pharmaceutical companies may use marketing tactics to promote the use of antibiotics for these conditions, even though they are not effective and may actually be harmful [24]. The overuse of antibiotics for non-bacterial conditions can contribute to the development of antibiotic resistance and expose patients to unnecessary risks, such as the side effects of these drugs or the development of resistant infections [25]. The ethical considerations surrounding the development and promotion of antibiotics by the pharmaceutical industry are complex and multifaceted. The profit motive of these companies and the potential conflicts of interest that may arise can influence the development and promotion of these drugs in ways that may not always prioritize the needs of patients or the public health implications of antibiotic resistance. Ensuring the availability and accessibility of essential antibiotics, while also promoting the responsible use of these drugs, is an important ethical consideration that requires the involvement of multiple stakeholders, including pharmaceutical industry.

Importantly, the role of R&D in addressing AMR is of paramount importance as the world faces increasing threats from resistant strains. However, ethical challenges arise in various aspects, such as resource allocation for R&D, the suitability of the patent-driven model, and collaboration between various stakeholders, including governments, academia, and the pharmaceutical industry. Tackling these challenges calls for a re-evaluation of the ethical dimensions surrounding resource allocation, incentives, and collaboration to ensure the development of new antibiotics and alternative treatments, ultimately mitigating the threat of AMR, and preserving the efficacy of life-saving antibiotics.

## The ethical dimension of resource transfer in addressing antimicrobial resistance

The burden of AMR is often disproportionately borne by developing countries with limited resources and weaker health systems. As such, there is an important ethical dimension to the question of resource transfer from the Global North to the Global South in the context of AMR. The establishment of a Global Fund on AMR, as proposed by Steven Hoffman et al., could aid to provide resources and support to developing countries in their efforts to combat AMR [26]. However, questions remain about the appropriate level of resource transfer and the ethical implications of such transfers. Should the Global North bear a greater share of the burden, given its historical responsibility for driving the development and spread of AMR? Or should the focus be on building the capacity of the Global South to tackle the problem themselves, with support from the international community?

Another ethical consideration is the potential for power imbalances and dependency relationships to arise from resource transfers. Developing countries may be in a weaker bargaining position when negotiating for resources or may be subject to conditions or strings attached to the aid they receive. This can create ethical challenges related to autonomy, agency, and justice. It is important to recognize and address these ethical dimensions of resource transfer in addressing AMR. Development assistance and similar types of support can play a crucial role in addressing the problem of AMR, but it is important to ensure that such efforts are equitable, just, and sustainable. The involvement and engagement of developing countries and their communities in the design and implementation of resource transfer initiatives can help to ensure that they are responsive to local needs and context and can also help to mitigate power imbalances and dependency relationships.

## Conclusion

Addressing the ethical dimensions of AMR requires a multifaceted approach that must consider various crucial factors. These factors include the ethical dimension of resource transfer in addressing AMR, which involves fair allocation of resources and promoting global equity. The environmental impact of antibiotic use, encompassing the need to mitigate pollution and ecological consequences resulting from the production, use, and disposal of antibiotics, is another significant consideration. The analysis should also cover conflicts of interest in the development and promotion of antibiotics by the pharmaceutical industry, ensuring that the public interest and patient needs take precedence over profit motives. Equitable access to antibiotics is a critical aspect, as it encompasses the need to guarantee that all individuals, regardless of their geographical location or socio-economic status, can access life-saving antibiotics. Lastly, stakeholder collaboration in antimicrobial stewardship is essential, involving the cooperation of governments, healthcare professionals, pharmaceutical companies, and the public in promoting responsible antibiotic use and developing innovative solutions to curb resistance. In a globalised world, balancing the risks and benefits of antibiotic use requires a comprehensive comprehension of the complex interplay between these factors and the implementation of innovative, collaborative strategies to preserve the efficacy of life-saving antibiotics for future generations.

## Data Availability

Not applicable.

## Abbreviations

AMR: Antimicrobial Resistance
WHO: World Health Organization
R&D: Research and Development
